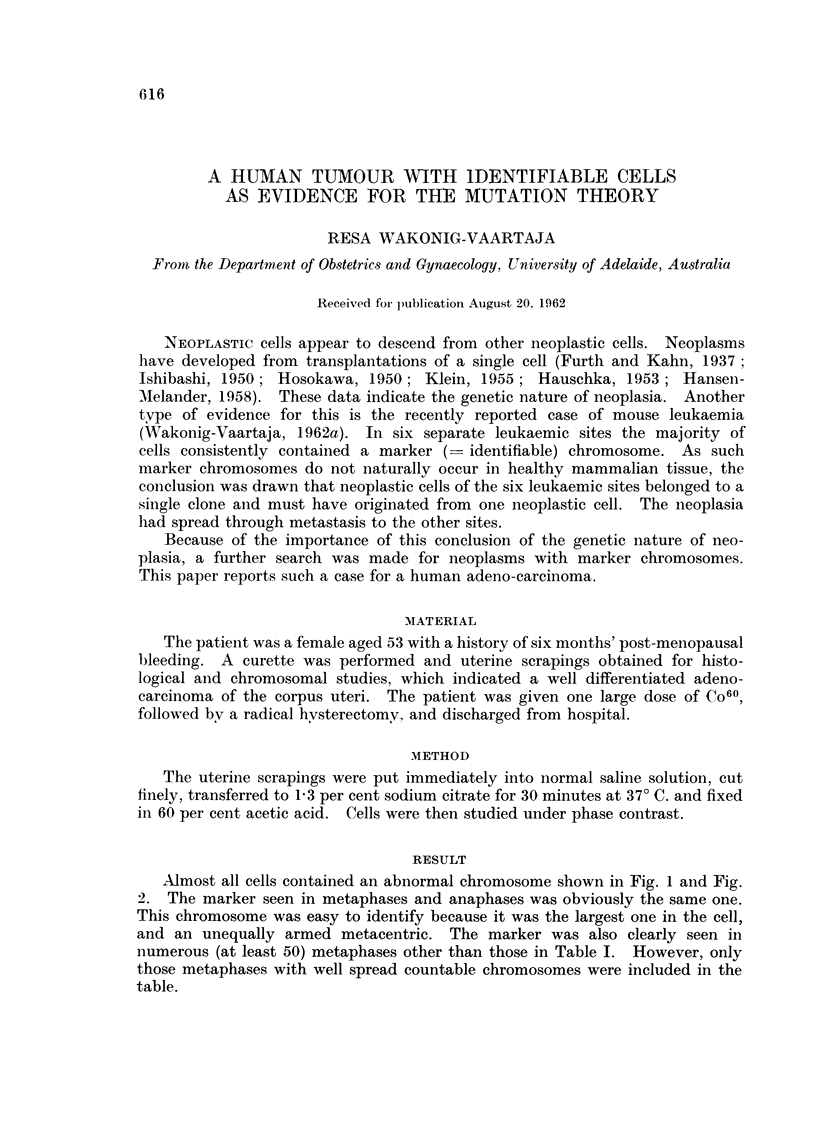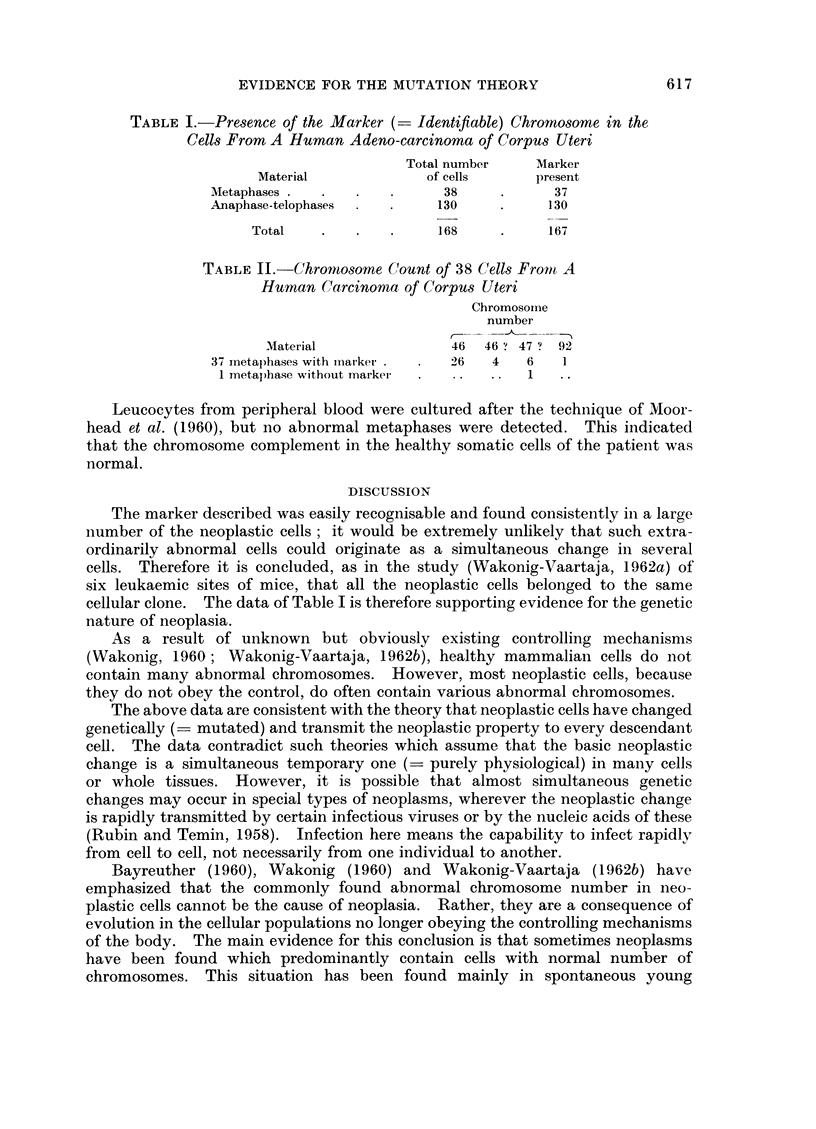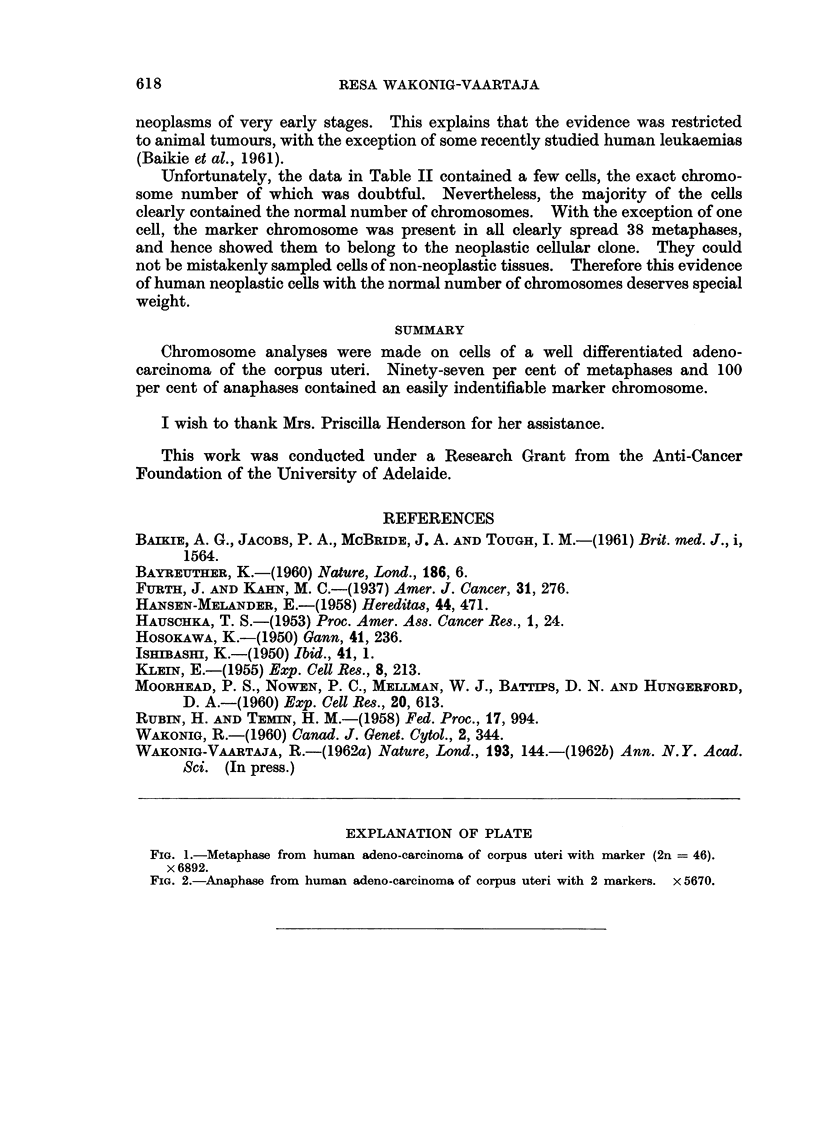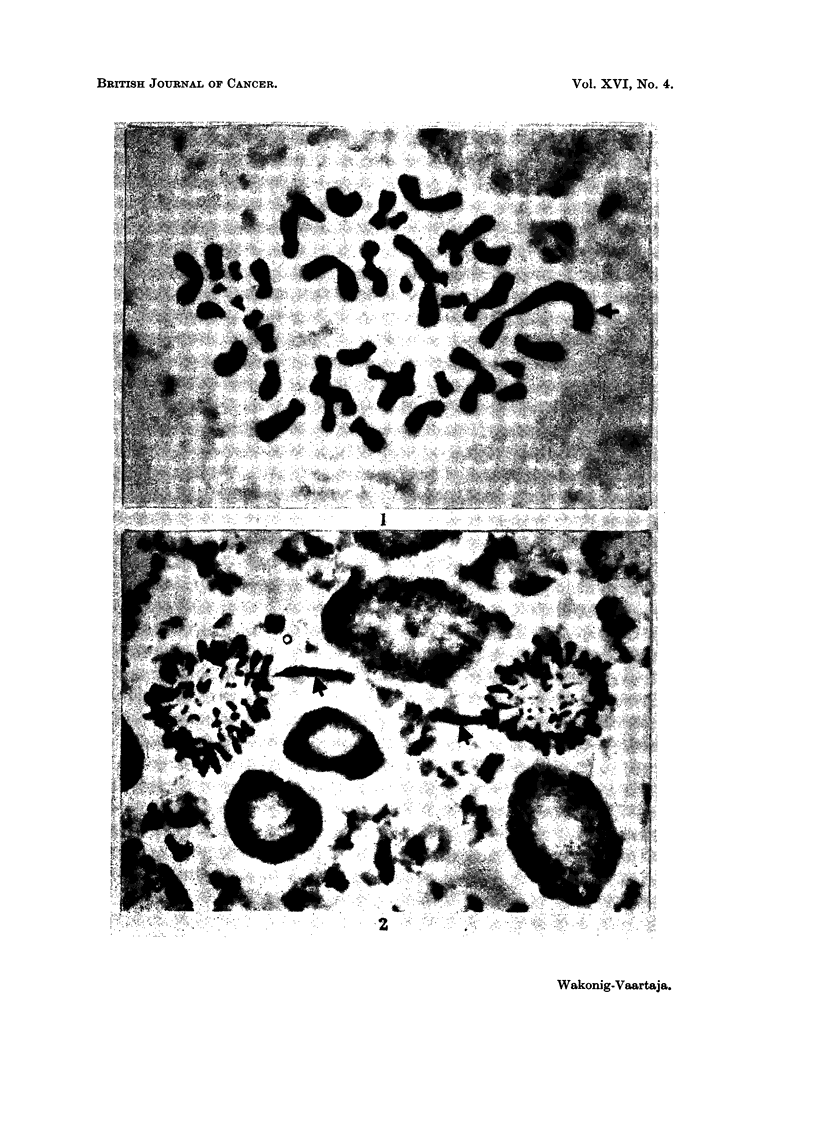# A Human Tumour with Identifiable Cells as Evidence for the Mutation Theory

**DOI:** 10.1038/bjc.1962.72

**Published:** 1962-12

**Authors:** Resa Wakonig-Vaartaja

## Abstract

**Images:**


### A HUMAN TUMOUR WITH IDENTIFIABLE CELLS AS EVIDENCE FOR THE MUTATION THEORY

RESA WAKONIG-VAARTAJA

*From the Department of Obstetrics and Gynaecology, University of Adelaide, Australia*

Received for publication August 20, 1962

NEOPLASTIC cells appear to descend from other neoplastic cells. Neoplasms have developed from transplantations of a single cell (Furth and Kahn, 1937; Ishibashi, 1950; Hosokawa, 1950; Klein, 1955; Hauschka, 1953; Hansen-Melander, 1958). These data indicate the genetic nature of neoplasia. Another type of evidence for this is the recently reported case of mouse leukaemia (Wakonig-Vaartaja, 1962*a*). In six separate leukaemic sites the majority of cells consistently contained a marker (= identifiable) chromosome. As such marker chromosomes do not naturally occur in healthy mammalian tissue, the conclusion was drawn that neoplastic cells of the six leukaemic sites belonged to a single clone and must have originated from one neoplastic cell. The neoplasia had spread through metastasis to the other sites.

Because of the importance of this conclusion of the genetic nature of neoplasia, a further search was made for neoplasms with marker chromosomes. This paper reports such a case for a human adeno-carcinoma.

### MATERIAL

The patient was a female aged 53 with a history of six months' post-menopausal bleeding. A curette was performed and uterine scrapings obtained for histological and chromosomal studies, which indicated a well differentiated adeno-carcinoma of the corpus uteri. The patient was given one large dose of $Co^{60}$, followed by a radical hysterectomy, and discharged from hospital.

### METHOD

The uterine scrapings were put immediately into normal saline solution, cut finely, transferred to 1·3 per cent sodium citrate for 30 minutes at 37° C. and fixed in 60 per cent acetic acid. Cells were then studied under phase contrast.

### RESULT

Almost all cells contained an abnormal chromosome shown in Fig. 1 and Fig. 2. The marker seen in metaphases and anaphases was obviously the same one. This chromosome was easy to identify because it was the largest one in the cell, and an unequally armed metacentric. The marker was also clearly seen in numerous (at least 50) metaphases other than those in Table I. However, only those metaphases with well spread countable chromosomes were included in the table.

TABLE I.—*Presence of the Marker (= Identifiable) Chromosome in the Cells From A Human Adeno-carcinoma of Corpus Uteri*

| Material | Total number of cells | Marker present |
|---|---|---|
| Metaphases | 38 | 37 |
| Anaphase-telophases | 130 | 130 |
| Total | 168 | 167 |

TABLE II.—*Chromosome Count of* 38 *Cells From A Human Carcinoma of Corpus Uteri*

| Material | Chromosome number | | | |
|---|---|---|---|---|
| | 46 | 46 ? | 47 ? | 92 |
| 37 metaphases with marker | 26 | 4 | 6 | 1 |
| 1 metaphase without marker | .. | .. | 1 | .. |

Leucocytes from peripheral blood were cultured after the technique of Moorhead *et al.* (1960), but no abnormal metaphases were detected. This indicated that the chromosome complement in the healthy somatic cells of the patient was normal.

### DISCUSSION

The marker described was easily recognisable and found consistently in a large number of the neoplastic cells ; it would be extremely unlikely that such extraordinarily abnormal cells could originate as a simultaneous change in several cells. Therefore it is concluded, as in the study (Wakonig-Vaartaja, 1962*a*) of six leukaemic sites of mice, that all the neoplastic cells belonged to the same cellular clone. The data of Table I is therefore supporting evidence for the genetic nature of neoplasia.

As a result of unknown but obviously existing controlling mechanisms (Wakonig, 1960 ; Wakonig-Vaartaja, 1962*b*), healthy mammalian cells do not contain many abnormal chromosomes. However, most neoplastic cells, because they do not obey the control, do often contain various abnormal chromosomes.

The above data are consistent with the theory that neoplastic cells have changed genetically (= mutated) and transmit the neoplastic property to every descendant cell. The data contradict such theories which assume that the basic neoplastic change is a simultaneous temporary one (= purely physiological) in many cells or whole tissues. However, it is possible that almost simultaneous genetic changes may occur in special types of neoplasms, wherever the neoplastic change is rapidly transmitted by certain infectious viruses or by the nucleic acids of these (Rubin and Temin, 1958). Infection here means the capability to infect rapidly from cell to cell, not necessarily from one individual to another.

Bayreuther (1960), Wakonig (1960) and Wakonig-Vaartaja (1962*b*) have emphasized that the commonly found abnormal chromosome number in neoplastic cells cannot be the cause of neoplasia. Rather, they are a consequence of evolution in the cellular populations no longer obeying the controlling mechanisms of the body. The main evidence for this conclusion is that sometimes neoplasms have been found which predominantly contain cells with normal number of chromosomes. This situation has been found mainly in spontaneous young neoplasms of very early stages. This explains that the evidence was restricted to animal tumours, with the exception of some recently studied human leukaemias (Baikie *et al.*, 1961).

Unfortunately, the data in Table II contained a few cells, the exact chromosome number of which was doubtful. Nevertheless, the majority of the cells clearly contained the normal number of chromosomes. With the exception of one cell, the marker chromosome was present in all clearly spread 38 metaphases, and hence showed them to belong to the neoplastic cellular clone. They could not be mistakenly sampled cells of non-neoplastic tissues. Therefore this evidence of human neoplastic cells with the normal number of chromosomes deserves special weight.

SUMMARY

Chromosome analyses were made on cells of a well differentiated adeno-carcinoma of the corpus uteri. Ninety-seven per cent of metaphases and 100 per cent of anaphases contained an easily indentifiable marker chromosome.

I wish to thank Mrs. Priscilla Henderson for her assistance.

This work was conducted under a Research Grant from the Anti-Cancer Foundation of the University of Adelaide.

### REFERENCES

BAIKIE, A. G., JACOBS, P. A., McBRIDE, J. A. AND TOUGH, I. M.—(1961) *Brit. med. J.*, i, 1564.
BAYREUTHER, K.—(1960) *Nature, Lond.*, **186**, 6.
FURTH, J. AND KAHN, M. C.—(1937) *Amer. J. Cancer*, **31**, 276.
HANSEN-MELANDER, E.—(1958) *Hereditas*, **44**, 471.
HAUSCHKA, T. S.—(1953) *Proc. Amer. Ass. Cancer Res.*, **1**, 24.
HOSOKAWA, K.—(1950) *Gann*, **41**, 236.
ISHIBASHI, K.—(1950) *Ibid.*, **41**, 1.
KLEIN, E.—(1955) *Exp. Cell Res.*, **8**, 213.
MOORHEAD, P. S., NOWEN, P. C., MELLMAN, W. J., BATTIPS, D. N. AND HUNGERFORD, D. A.—(1960) *Exp. Cell Res.*, **20**, 613.
RUBIN, H. AND TEMIN, H. M.—(1958) *Fed. Proc.*, **17**, 994.
WAKONIG, R.—(1960) *Canad. J. Genet. Cytol.*, **2**, 344.
WAKONIG-VAARTAJA, R.—(1962*a*) *Nature, Lond.*, **193**, 144.—(1962*b*) *Ann. N.Y. Acad. Sci.* (In press.)

---

EXPLANATION OF PLATE

FIG. 1.—Metaphase from human adeno-carcinoma of corpus uteri with marker ($2n = 46$). ×6892.

FIG. 2.—Anaphase from human adeno-carcinoma of corpus uteri with 2 markers. ×5670.